# Selected imprinting of *INS* in the marsupial

**DOI:** 10.1186/1756-8935-5-14

**Published:** 2012-08-28

**Authors:** Jessica M Stringer, Shunsuke Suzuki, Andrew J Pask, Geoff Shaw, Marilyn B Renfree

**Affiliations:** 1 ARC Centre of Excellence in Kangaroo Genomics, University of Melbourne, Melbourne, Victoria, 3010, Australia; 2 Department of Zoology, The University of Melbourne, Melbourne, Victoria, 3010, Australia; 3 Epigenomics Division, Frontier Agriscience and Technology Center, Faculty of Agriculture, Shinshu University, Nagano, 399-4598, Japan; 4 Department of Molecular and Cellular Biology, The University of Connecticut, Storrs, Connecticut, CT06269, USA

**Keywords:** Genomic imprinting, Mammary gland, Lactation, Marsupial, Insulin, Co-adaptation

## Abstract

**Background:**

In marsupials, growth and development of the young occur postnatally, regulated by milk that changes in composition throughout the long lactation. To initiate lactation in mammals, there is an absolute requirement for insulin (*INS*), a gene known to be imprinted in the placenta. We therefore examined whether *INS* is imprinted in the mammary gland of the marsupial tammar wallaby (*Macropus eugenii*) and compared its expression with that of insulin-like growth factor 2 (*IGF2*).

**Results:**

*INS* was expressed in the mammary gland and significantly increased, while *IGF2* decreased, during established milk production. Insulin and IGF2 were both detected in the mammary gland macrophage cells during early lactation and in the alveolar cells later in lactation. Surprisingly, *INS*, which was thought only to be imprinted in the therian yolk sac, was imprinted and paternally expressed in the liver of the developing young, monoallelically expressed in the tammar mammary gland and biallelic in the stomach and intestine. The *INS* transcription start site used in the liver and mammary gland was differentially methylated.

**Conclusions:**

This is the first study to identify tissue-specific *INS* imprinting outside the yolk sac. These data suggest that there may be an advantage of selective monoallelic expression in the mammary gland and that this may influence the growth of the postnatal young. These results are not consistent with the parental conflict hypothesis, but instead provide support for the maternal–infant co-adaptation hypothesis. Thus, imprinting in the mammary gland maybe as critical for postnatal growth and development in mammals as genomic imprinting in the placenta is prenatally.

## Background

Genomic imprinting is an epigenetic modification to the DNA that regulates the expression of selected genes from only one parental allele. In vertebrates, imprinting is restricted to the therian (marsupial and eutherian) mammals but as yet no imprinted genes have been identified in monotremes [1,2]. Thus mammalian genomic imprinting is thought to have evolved after the therian–monotreme divergence. In mice and humans, most imprinted genes are expressed in the placenta, some of which are exclusively imprinted in this organ [3-7]. Although the significance of imprinted gene expression is still debated, many imprinted genes regulate growth and nutrient provisioning to the developing fetus [8-11]. Potentially, therefore, any organ that regulates growth via nutrient exchange with the developing young may have imprinted gene expression. Imprinted gene expression occurs in the hypothalamus to regulate maternal behaviour, metabolism and milk letdown [12-14]. Paternally expressed gene 3 (*Peg3*) and MAGE-like gene 2 are examples of genes that are imprinted in the neonatal hypothalamus. Neonatal *Peg3* knockout mice lose their ability to find the teat and feed, while MAGE-like gene-2-deficient mice markedly reduce their activity, metabolism and food intake [13,15,16]. In the adult, heterozygous *Peg3* knockout mothers have impaired milk letdown and fail to allow sucking by the pups while heterozygous males have naive sexual behaviour [12-14,17-19]. Paternally expressed gene 1 (*Peg1*) deficient females have abnormal maternal behaviour and impaired placentophagia, sometimes leaving their pups untouched after parturition [20]. A large number of autosomal genes with sex-specific imprinting in the cortex and hypothalamus have been identified recently, but as yet there are no data on their possible functions [21,22].

Mammary glands, lactation, maternal and neonatal behaviour involved in postnatal feeding are all essential mammalian characteristics that regulate and enhance the growth and survival of young. The mouse X chromosome is nonrandomly maternally inactivated in mammary epithelial cells [23]. In the same study, the X-linked gene Rnf12, which encodes the ubiquitin ligase Rnf12/RLIM, is identified to be a critical survival factor for milk-producing alveolar cells [23]. Limited breast cancer studies have similarly demonstrated monoallelic expression in the mammary gland [24-26]. For example, there is monoallelic expression of insulin-like growth factor 2 (*IGF2*) in normal breast tissue and in all but two cases of breast cancer [27]. These data indicate that certain growth and survival factors are selectively monoallelically expressed in the eutherian mammary gland.

*INS* encodes insulin, a polypeptide hormone that regulates carbohydrate metabolism, cell growth and survival, protein synthesis, vascularisation and vasodilation [28-31]. Alternative *INS* transcripts, with either an extended 5^′^UTR or a retained intron, and chimaeric transcripts with exons from the upstream tyrosine hydroxylase(*TH*) gene have been identified in the developing chick*.* These alternative transcripts all result in decreases in the efficiency of protein translation, which may control cell survival in the early developing embryo [32-35].

Insulin is essential for the induction of milk protein synthesis in mammals [36-41]. Elevated insulin levels in the goat mammary gland increase milk production and the milk protein content [42]. Similarly in dairy cows, the milk protein yield increases by approximately 15% after insulin administration [43,44]. *INS* is located upstream of the growth-promoting, paternally expressed gene *IGF2*, and functions in the mammary gland in conjunction with *IGF2* to induce growth and alveologenesis [38]. *IGF2* was the first imprinted gene identified in both eutherians and marsupials [45,46]. In eutherians, *IGF2* is imprinted in most fetal and adult tissues –but in the marsupial, although the maternal *IGF2* allele is completely silenced in the fetal and pouch young liver, silencing is incomplete in the placenta and *IGF2* is biallelically expressed in the adult liver [47,48]. In the eutherian mammary gland, *IGF2* acts with cyclin D_1_ to mediate the prolactin-induced proliferation of mammary epithelial cells during alveolar formation [38,49].

Insulin and IGF2 are both found in the milk of humans, dairy cows and rats [50-52]. They are present at the highest concentrations in colostrum, but are still present at low concentrations in milk produced later in lactation [52]. Milk IGFs may support nutrient transfer to the young by increasing mucosal cell turnover and enhancing villus growth of the newborn’s gastrointestinal tract [51,52].

*INS* imprinting has been analysed previously, but only in the pancreas and the yolk sac of mice and humans. Paternal expression, and therefore imprinting, is detected only in the yolk sac, but there is biallelic expression in the pancreas and fetal head and body and so it is not imprinted in these tissues [53-56]. Similarly, there is paternal *INS* expression in the tammar wallaby (*Macropus eugenii*) yolk sac placenta [57]. Imprinting of the mouse *Ins*2 (the homologue of human *INS*) gene and the *Igf2* gene is disrupted by maternal inheritance of a targeted deletion of the *H19* gene and its flanking sequence, so imprinting of *INS* is regulated by the same imprinting control region as *IGF2* and *H19*[58].

The identification of *Peg3*, a hypothalamic gene that regulates maternal suckling behaviour in mice, prompted the development of the maternal–infant co-adaptation hypothesis [14,19,59] as an alternative to the parental conflict hypothesis to explain the evolution and maintenance of genomic imprinting in mammals [60,61]. The conflict hypothesis predicted that imprinting in the fetus and placenta evolved as a consequence of competition between the male and female genomes to optimise their respective reproductive success [60]. In contrast, the maternal–infant co-adaptation hypothesis predicts that imprinting evolved to enhance the genetic integration of the intimate maternal–offspring interactions [19,59]. For example, genes in the offspring that regulate the offspring’s requirements and behaviour (for example, nutritional demand and sucking) and genes in the mother that regulate her response (for example, nutritional supply and suckling) may acquire tissue-specific imprinted expression (for example, placenta, mammary gland and brain) to allow a greater potential for rapid fixation of beneficial traits in the population [12,13,19,59,62]. This hypothesis provides a clear explanation for the presence of imprinting before and after birth and could apply to genes expressed in the mammary gland that regulate both maternal milk production and supply. *INS*, which is imprinted in the placenta, is also important for the development and function of the mammary gland, and so there may be imprinted expression in this unique mammalian organ to regulate nutrient production and transport to the neonate after birth as the placenta does before birth*.*

To examine this hypothesis we investigated the allelic expression of *INS* in the marsupial mammary gland. Marsupials give birth at a much earlier developmental stage than most eutherians and have only a short-lived chorio-vitelline placenta. The vast majority of growth and development of the young occurs postnatally through a long, complex and physiologically sophisticated period of lactation [63,64]. In the tammar the length of lactation is 9 months, during which the young increase from a birth weight of ~450 mg to ~2.5 kg before they are fully weaned. Three broad phases can be recognised, during which the size of the mammary gland and the composition of the milk change. In the tammar, as in other marsupials, milk composition changes in concert with the developmental stage of the young to directly regulate its growth. Developmental acceleration or retardation can be experimentally induced when young are reared on either late-stage or early-stage milk, respectively [65-67]. Thus the mammary gland is an obvious target for imprinting under the maternal–infant co-adaptation hypothesis. Furthermore, both mammary gland size (correlated to the volume of milk produced) and the composition of the milk could potentially be regulated by both *INS* and *IGF2*. We hypothesised that if imprinting enhances the production and transport of nutrients and growth factors, as the co-adaptation hypothesis predicts, the mammary gland, particularly in marsupials, would be a primary site of imprinted gene expression. We examined this hypothesis by analysing the allelic expression of *INS* and compared it with that of *IGF2* in the tammar wallaby throughout lactation.

## Results

### Characterisation of *INS* and *IGF2* mRNA expression in the tammar mammary gland

**Figure 1:**
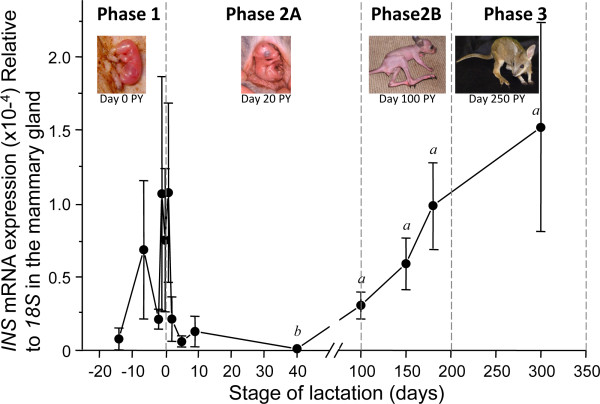
*** INS*mRNA expression relative to*18S*mRNA expression in the mammary gland.***INS* mRNA expression (mean ± standard error of the mean × 10^-4^) compared with*18S* expression in the mammary gland across the four phases of lactation. After birth, *INS* remained low until after 100 days of lactation.*a* significantly higher than *b* (*P* < 0.05). *INS*, insulin gene.

*INS* and *IGF2* were expressed throughout the three broad phases of lactation: phase 1 (during pregnancy), phase 2A (day 0 to day 100 of lactation), phase 2B (day 100 to day 200 of lactation) and phase 3 (day 200 to day 350 of lactation). There was no statistically significant variation in *INS* expression throughout the early stages of lactation, before day 5, when the gland is developing and producing colostrum. However, there was a significant increase in *INS* expression during established milk production between day 9 and day 300 of lactation (*P* < 0.05) (Figure 1).

**Figure 2:**
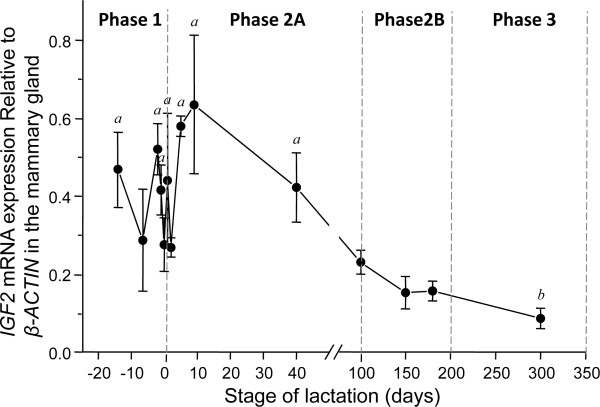
*** IGF2*mRNA expression relative to*β-ACTIN*mRNA expression in the mammary gland.***IGF2* mRNA expression (mean ± standard error of the mean) compared with *β-ACTIN* expression in the mammary gland across the four phases of lactation. *IGF2* was highest in the perinatal period, but steadily decreased throughout the remainder of lactation. *a* significantly higher than *b*(*P* < 0.05). IGF, insulin-like growth factor.

*IGF2* expression was high during early lactation and decreased from phase 2 onwards (*P* < 0.05) (Figure 2). There was a significant negative correlation (*P* < 0.05) between *INS* and *IGF2* after day 5 of lactation.

### Localisation of insulin and IGF2 protein in the tammar mammary gland

**Figure 3:**
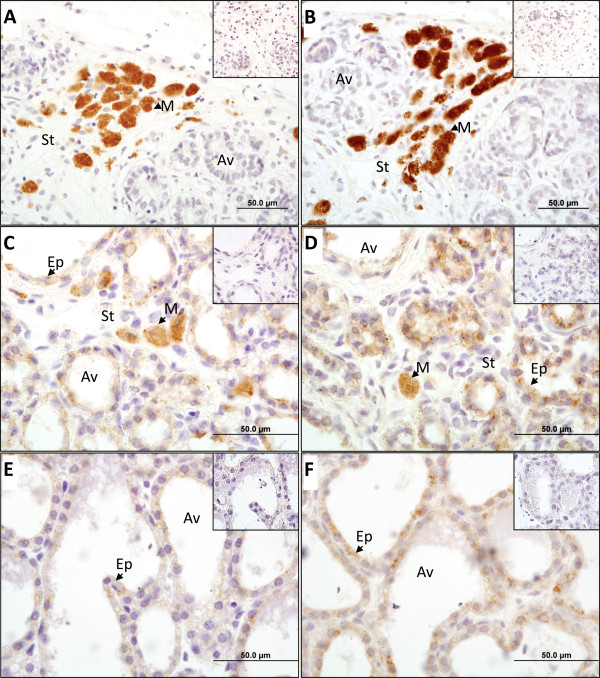
** Immunolocalisation of insulin and IGF2 protein in the tammar mammary gland.** Insulin (**A**), (**C**) and (**E**) and insulin-like growth factor 2 (IGF2)**(B)**, **(D)** and **(F)** protein (brown staining) localisation in mammary glands at three different stages of lactation. (**A**), (**B**) One day before birth (day −1), macrophage cells (M) in the stroma (S) were intensely stained and there was no staining in the alveolar cells (Av). (**C**), (**D**) At day 95 of lactation, insulin and IGF2 was detected in the cytoplasm of the alveoli epithelial cells (Ep), with infrequent macrophage cell staining. (**E**), (**F**) At day 200 of lactation, staining was concentrated in the epithelial cells of the alveoli. There was no staining in any of the IgG antibody control (top right insert). There was no staining in any of the no-antibody controls (data not shown). Scale bars shown at the bottom right.

Both insulin and IGF2 were strongly stained and localised in the cytoplasm of macrophage cells in the stromal cells during lactogenesis phase 1 (Figure 3A,B). At later stages, protein was detected predominantly in the cytoplasm of the alveolar epithelial cells in the lactating mammary gland, with some macrophages containing both insulin (Figure 3C,E) and IGF2 (Figure 3D,F). There was no cytoplasmic or nuclear staining in the IgG antibody controls (Figure 3, insets) or in the no-antibody (diluent-only) negative controls. The cytoplasmic location of insulin in the tammar placenta was similar to that previously reported [57]. In the pancreas, the insulin antibody was detected only in the cytoplasm of the cells in the islets of Langerhans (see Additional file 1). 

### Monoallelic expression in the tammar mammary gland

#### The *INS* gene

**Figure 4:**
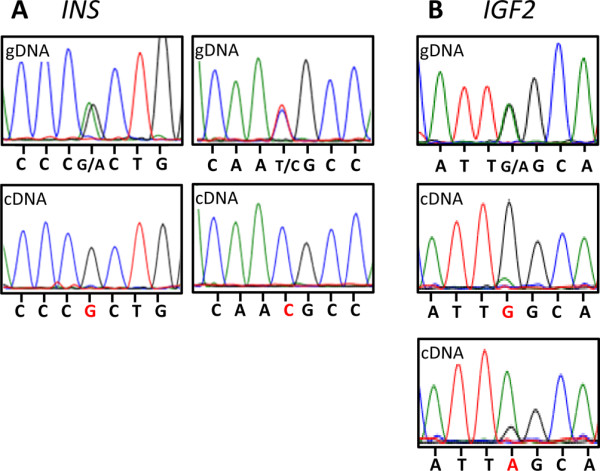
** Sequence chromatographs for imprint analysis in the mammary gland.** Direct sequence analysis for insulin (*INS*) and insulin-like growth factor 2 (*IGF2*) imprinting in the mammary gland. (**A**) *INS* chromatogram traces (viewed in Finch TV version 5.1) for genomic DNA (gDNA) and cDNA. Two polymorphic sites were identified in gDNA, distinguishing the two alleles. In the mammary gland of seven individuals tested, two animals had monoallelic expression at both SNPs (site 1:G/A and site 2: C/T) and five animals had monoallelic expression in a single SNP (one individual at site 1and four individuals at site 2). (**B**) *IGF2* chromatogram traces for gDNA (3^′^ to 5^′^) and cDNA (5^′^ to 3^′^). A single polymorphic site was identified in gDNA to distinguish the two alleles. Both alleles of *IGF2* were monoallelically expressed in four different individuals (three animals expressed the G allele and one animal the A allele).

Two previously identified *INS *SNPs located 16 base pairs apart [57] were used to identify the imprint status of *INS*. Of 20 adult individuals, seven animals were polymorphic (two animals were polymorphic at both SNP sites, one animal was polymorphic at site 1 and four animals were polymorphic at site 2). cDNA amplified from the mRNA of all seven polymorphic individuals showed monoallelic expression (Figure 4A). A population of 32 animals were genotyped for the two SNPs and it was concluded that three different *INS* alleles (G-C, G-T and A-T) exist in the tammar population (see Additional file 2: Tables S1 and S2). Of these three alleles, allele 1 (G-C) and allele 2 (G-T) were monoallelically expressed in the mammary gland (see Additional file 2: Table S3). 

**Figure 5:**
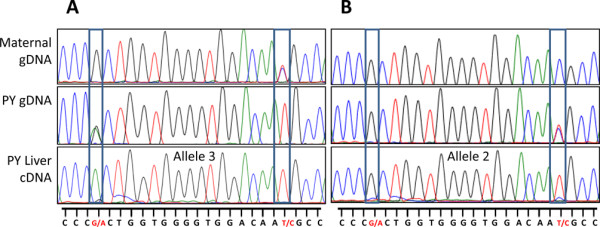
*** INS*sequence chromatographs for imprint analysis in the pouch young liver.** Direct sequence analysis for *INS* in the pouch young liver. Chromatogram traces of genomic DNA (gDNA) from the mother and pouch young and of cDNA from the pouch young liver. (**A**) The pouch young inherited allele 2 (G-T) from its mother, and the clear monoallelic expression of allele 3 (A-T) in the liver was inherited from the father. (**B**) The pouch young inherited allele 1 (G-C) from its mother, and the clear monoallelic expression of allele 2 (G-T) in the liver was inherited from the father. RNA was extracted twice from the same liver sample and direct sequencing produced the same results in both samples. These results indicate that *INS* expression in the liver is a result of parent-of-origin specific genomic imprinting and not random monoallelic expression. *INS*, insulin gene.

To determine whether *INS* monoallelic expression was a global phenomenon in the tammar or was selectively maintained in the mammary gland, the allelic expression of *INS* was analysed in various adult and pouch young tissues. cDNA was amplified from four different tissues from five pouch young, all heterozygous at the identified SNPs and between the age of 10 and 100 days old. Biallelic expression was detected in the stomach and intestine, biased expression was observed in the adult adrenal gland and monoallelic expression was observed in nine out of 10 livers (three out of three adults and six out of seven pouch young) examined (Table 1). All three alleles were monoallelically expressed in the pouch young liver: two pouch young expressed allele 1, two pouch young expressed allele 2 and two pouch young expressed allele 3 (A-T). Two pouch young with known maternal genotype showed only paternal *INS* expression of allele 3 in the liver in two separate RNA extractions (Figure 5A). A third pouch young showed paternal expression of allele 2 in the liver (Figure 5B).

**Table 1:** **Allelic expression of*INS*in different adult and pouch young tammar tissues**

**Sex**	**Age (days)**	**Brain**	**Stomach**	**Intestine**	**Liver**	**Adrenal gland**
F	15 to 16	>	Absent	Biallelic	Monoallelic (allele 1)	nd
M	30	nd	nd	nd	Monoallelic (Pat, allele 2)	nd
M	36	>	Biallelic	Biallelic	Monoallelic (allele 1)	nd
M	45 to 46	Low	Biallelic	Biallelic	Biallelic	nd
M	52	Low	Biallelic	Biallelic	Monoallelic (allele 2)	nd
M	81	Low	Biallelic	Biallelic	Monoallelic (Pat, allele 3)	nd
F	182	nd	nd	nd	Monoallelic (Pat, allele 3)	nd
F	Adult	nd	nd	nd	Monoallelic (allele 2)	>
F	Adult	nd	nd	nd	Monoallelic (allele 1)	>
F	Adult	nd	nd	nd	Monoallelic (allele 1)	>

Pouch young and adult tissues analysed for *INS* imprinting via direct sequencing. Both female (F) and male (M) samples had monoallelic, biallelic or biased (>) expression. Some tissues had very low RT-PCR amplification (low) or no amplification (absent) so imprinting could not be determined. Three pouch young with a known maternal genotype showed paternal (Pat) monoallelic expression in the liver. nd, not determined.

#### The *IGF2* gene

Of 20 individuals analysed, seven animals were polymorphic at one of the previously identified *IGF2* SNP sites in the tammar [68]. Monoallelic expression was observed in the mammary gland from two different *IGF2* alleles (Figure 4B).

#### The *INS* transcription start site was not differentially methylated in the mammary gland

**Figure 6:**
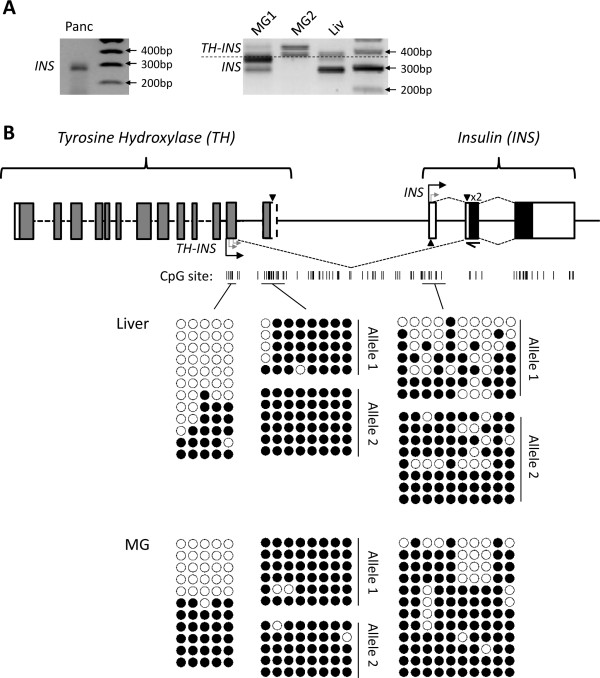
** Structure and methylation of tammar*INS.*** (**A**) 5^′^-Rapid amplification of cDNA ends (5^′^-RACE) was performed on RNA derived from one pancreas (Panc), two mammary glands (MG) and one liver (Liv). Five *INS* transcripts were amplified using a primer designed in the first *INS* coding exon (half-arrow). Three transcripts were chimeras and contained an exon derived from the neighbouring tyrosine hydroxylase (*TH*) gene and two were transcribed from the *INS* noncoding exon. The mammary gland 1 (MG1; lactation phase 1) and liver expressed both types of transcripts, the pancreas expressed only the INS-derived transcripts, and the mammary gland 2 (MG2; lactation phase 3) expressed only the *TH-INS* transcripts. (**B**) Schematic of predicted tammar *TH* and *INS* genes (not to scale). Predicted coding exons (grey), verified coding exons (black) and noncoding exons (white) are represented by boxes. Transcription start sites identified by 5^′^-RACE are indicated with turned arrows. CpGs are indicated by short vertical black lines. SNPs are indicated by black triangles. Bisulphite sequenced regions (black horizontal lines) are shown with individual bisulphite sequences underneath: open and closed circles are unmethylated and methylated CpGs, respectively. Each row represents the methylation pattern on a separate DNA fragment from the same sample. Both methylated and unmethylated alleles were present in the liver and mammary gland tissues at the *TH-INS* TSS. Only methylated alleles were present at the CpG Island and the *INS* TSS had a variable methylation pattern.*INS*, insulin gene.

5^′^-Rapid amplification of cDNA ends (5^′^-RACE) was used to identify the transcription start sites (TSSs) of *INS*. A single transcript was identified in the adult pancreas, while multiple transcripts were identified in the mammary gland and liver (Figure 6A). TSSs were located in the *INS* first exon and in the second to last predicted exon of the tammar *TH* gene (Figure 6B), separated by approximately 3.6 kb. Only *INS* transcripts were identified in the pancreas whereas both *INS* and *TH-INS* transcripts were detected in the mammary gland and liver (Figure 6A). Repeated imprint analysis, using transcript-specific primers, showed conserved paternal expression of *TH-INS* in two pouch young livers, and conserved monoallelic expression of *INS* in two mammary glands. The genomic region around the *TH-INS* and *INS* TSSs were relatively CpG-rich, and a CpG island (CGI) was located downstream of the *TH-INS* TSS, at the location of the predicted last *TH* exon (Figure 6B). The methylation status of these three regions was analysed by bisulphite sequencing, using primers designed by MethPrimer [69], to determine whether *INS* allelic expression was regulated by differential methylation. Methylation was assessed in both liver and mammary tissue (Figure 6). A SNP was located in the CGI and near the *INS* TSS. These were used to distinguish between alleles in heterozygous samples. The *TH-INS* TSS appears to have a differentially methylated region (DMR)-like methylation pattern, but no usable SNP was identified within this region to determine allele specificity of the methylation. No allele-specific methylation was observed at the *INS* TSS and the CGI was completely methylated (Figure 6). 

## Discussion

The tissue-specific monoallelic expression of *INS* in the marsupial mammary gland suggests that there may be a role for genomic imprinting during lactation. Marsupials have a long and physiologically sophisticated lactation that provides fine control of the growth and development of the marsupial young by the mother. Thus imprinting in the mammary gland could regulate postnatal growth in mammals, just as imprinting in mammalian placenta regulates prenatal growth and development.

Both *INS* and *IGF2* were expressed in mammary glands throughout lactation, but the expression patterns differed. The increase in *INS* mRNA between phase 2A and phase 3 may reflect a role for endogenous insulin in the hyperplasia that characterises late lactation. This increase also coincides with a marked increase in volume and a change in milk composition. Insulin is required for the initiation of lactation in mammals and induces casein gene expression [36,41,43]. More specifically, insulin plays a crucial role in the transcription of at least 18 milk genes including *Stat5a* and *Elf5*, two major milk protein gene transcription factors and key components of prolactin signalling [40]. The increase of *INS* expression is therefore consistent with it regulating the increase in volume and the change to mature milk in the mammary gland. Relative *IGF2 *mRNA expression was highest in phase 1, consistent with paracrine signalling of *IGF2* regulating the initial proliferation of mammary gland epithelial cells [49,70].

During phase 1 of lactation, insulin and IGF2 protein were localised in the stroma, specifically in the macrophages. Macrophages are critical for normal mammary gland branching and alveolar morphogenesis [71,72]. Insulin and IGF2 may therefore regulate epithelial cell proliferation and morphogenesis as the mammary gland prepares for lactation [38]. Later in lactation, both insulin and IGF2 were detected in the cytoplasm of the epithelial cells of the alveoli, so it is likely that both proteins are secreted into the tammar milk, as in the cow and rat [51,52].

### Is *INS* imprinted in the marsupial mammary gland?

In the tammar, there was clear monoallelic *INS* expression in the mammary gland of seven individuals from phase 1 through to phase 2B of lactation. As reported previously in the tammar yolk sac placenta [57], *INS* was paternally expressed and therefore imprinted in the pouch young liver. *INS* was biallelically expressed in both the stomach and intestine and was biased in the adrenal gland, demonstrating that *INS* monoallelic expression is not widespread but is specifically maintained in the marsupial liver, mammary gland and placenta. As most specimens were collected from the wild, the parental genotypes were unknown. Because paternal expression of *INS* was established in the liver (Figure 5) and previously in the yolk sac [57] and we detected monoalleleic expression of three different *INS* alleles in the mammary gland and liver and a differentially methylated TSS, our data suggest that *INS* monoallelic expression is most probably due to genomic imprinting and not due to random monoallelic expression or allelic difference [73]. However, this cannot be definitively proven without a reciprocal cross. Based on limited analyses in mice and humans, the *INS* gene was presumed to be exclusively imprinted in the yolk sac [55,74]. Interestingly, there is an alternatively spliced *INS-IGF2* transcript that is imprinted and paternally expressed in the human fetal eye and limb [75]. *INS* monoallelic expression may therefore exist in tissues other than the yolk sac in eutherians, as it does in marsupials.

The marsupial mammary gland is the primary site for nutrient exchange between mother and infant. Our results suggest that *INS* may directly initiate and maintain alveologenesis and play a part in controlling the change in tammar milk composition by up regulating major milk protein gene transcription and translation factors [40,41]. As *INS* is critical for mammalian lactation, imprinting may have been maintained in this tissue to control mammary gland development and the transcription of vital milk proteins. Such a role is unlikely to be restricted to marsupials since eutherian mammals also depend on lactation to support their developing young after birth.

### Methylation of the transcription start site may regulate the expression of *INS*

The methylation pattern seen at the *TH-INS* TSS strongly implies that this is a differentially methylated region, although this remains to be confirmed. Tammar *INS* expression could be regulated by the conserved differentially methylated imprinting control region located between *IGF2* and *H19*[47], which regulates *INS* imprinting in both humans and rodents [58,76]. The putative DMR located at the *THINS* TSS may regulate imprinting in a similar manner to the DMRs located at each of the human *IGF2* promoters [77]. The high level of methylation at the CGI may be in place to prevent the inclusion of the last *TH* exon into the *TH-INS* mRNA. The variable methylation pattern observed at the *INS* TSS suggests that methylation at this TSS could regulate tissue-specific expression in the tammar as it does in eutherians [78]. *INS* imprinting could be transcript specific, as it is in the mouse imprinted gene Dopa decarboxylase [79] and the human imprinted *GRB10*[80] and *IGF2*[77,81] genes. However, preliminary analysis indicates that both *INS* and *TH-INS* transcripts are monoallelically expressed. *INS *may be the fourth marsupial imprinted gene to be associated with a DMR [47,82,83] and, if so, provides further evidence of a common origin of imprinting mechanisms in therian mammals.

## Conclusions

Both *INS* and *IGF2* are imprinted and paternally expressed in the marsupial placenta and in the liver (this study) [57,68]. Both genes are also monoallelically expressed in the mammary gland, suggesting that both may be imprinted in this tissue. The differential methylation of the *TH-INS* TSS in both the liver and mammary gland strongly supports the suggestion that *INS* is also imprinted in the mammary gland. This is the first indication that genomic imprinting may occur in a marsupial mammary gland and is consistent with the predictions of the maternal–infant co-adaptation hypothesis that genomic imprinting is involved in regulating the growth and development of young postnatally. *INS* was biallelically expressed in the developing digestive tract. Retention of monoallelic expression of *INS* therefore appears to be under selection in the mammary gland as well as in the placenta and liver. The mammary gland is a unique mammalian organ that regulates postnatal nutritional transfer by a positive feedback loop with the mother’s brain in response to the sucking stimulus. This interaction is similar to that observed between the placenta, fetus and the maternal hypothalamus. Genomic imprinting in the mammary gland may therefore be as critical for regulating postnatal growth as it is for regulating prenatal growth in the placenta.

## Methods

All experiments and wild animal collection were approved by the University of Melbourne Animal Experimentation Ethics Committee, and the animal handling and husbandry procedures were in accordance with the National Health and Medical Research Council of Australia 2004 guidelines. Animals were collected under approval from the South Australian Department of Environment and Natural Resources.

### Animals

Tammar wallabies of Kangaroo Island, South Australia origin were held at our colony in Melbourne. Pregnancy was initiated in females carrying an embryo in diapause by the removal of their pouch young [63]. Adult females carrying fetuses in the final third of gestation (day 19 to day 26 of the 26.5 day pregnancy) or pouch young (day 0 to day 350 postpartum) were killed either by shooting in the wild, stunning and cervical dislocation or by an anaesthetic overdose (sodium pentobarbitone, 60 mg/ml to effect). The lactating mammary gland and adult and pouch young tissues were collected and snap frozen in liquid nitrogen. As the animals were collected from the wild, the parental genotypes for most samples were unknown. Tammar wallabies are seasonal breeders, and males do not reach sexual maturity until approximately 2 years of age and females are unreliable breeders until they attain at least 2 years of age [63]. In addition, all females carry a diapausing blastocyst conceived a year before the birth of the new young, so it is extremely difficult to determine paternity in the wild population. Executing a targeted mating between homozygous individuals and producing an informative heterozygous adult lactating female was therefore beyond the scope of the present study.

### RNA and DNA extraction and RT-PCR

Individual genotypes were identified using PCR and direct sequencing of genomic DNA extracted from approximately 20 mg snap-frozen tissue using a Wizard Genomic DNA Purification Kit (Promega, Madison, WI, USA). Total RNA was extracted from mammary glands using the RNeasy Lipid Tissue Mini Kit (Qiagen,Hilden, Germany) or from other tissues using Tri-Reagent (Ambion, Austin, Texas, USA) as described by the manufacturer with a final elution of RNA in 60 to 80 μl RNAsecure H_2_O (a dilution of 1/24 μl RNAsecure to water;Ambion). Total RNA was DNase treated (DNA-free™; Ambion) to remove contaminating DNA, run on a 1% agarose gel to assess the quality, quantified with a nano-spectrometer (NanoDrop ND-1000 Spectrophotometer; NanoDrop Technologies Inc., Wilmington, DE, USA) and cDNA was synthesised using the SuperScript III First Strand Synthesis System for RT-PCR (Invitrogen, Carisbad, CA, USA). Typically, 2 μg or a maximum of 8 μl total RNA was used in each cDNA synthesis reaction, with 1 μl Oligo (dT)12-18 (50 μM). cDNA integrity was immediately assessed with *GAPDH* RT-PCR. All primers were designed using Primer3 (v. 0.4.0) [84] and were synthesised by Sigma-Aldrich (Castle Hill, NSW, Australia) (see Additional file 3).

### Immunohistochemistry

Insulin and IGF2 protein distribution was assessed in 10 mammary glands: four glands from phase 1 of lactation (day 25 of pregnancy, 1 day before birth), and six glands from phase 2 (three each from days 100 and 200 of lactation,post partum). IGF2 immunohistochemistry was performed as previously described [85] (Sc-7435; Santa Cruz, Santa Cruz, CaliforniaUSA). Insulin B N-20(sc-7838; Santa Cruz) antibody, raised against an epitope with 88% identity (15/17) to the tammar insulin predicted protein, was used to assess insulin distribution. Pancreas and placental tissues were used as controls to assess specificity of the antibody (see Additional file 1). The immuno histochemistry protocol was optimised in the mammary gland for both antibodies. Small pieces of mammary gland (placenta and pancreas for positive controls) were fixed in 4% paraformaldehydebefore paraffin embedding. Sections (6 to 7 μm) were mounted onto polylysine-coated slides (polysine;Menzel-Glaser; Lomb Scientific, Thermo Fisher, Waltham, MA, USA) before dewaxing, rehydrating and washing in 0.1% Triton-X-100. A 10-minute incubation in 0.05% Pronase (type XXIV; Sigma-Aldrich) preceded blocking in 10% normal rabbit serum/Tris-buffered saline/0.1% BSA. Primary antibodies (Insulin B N-20,sc-7838 at 0.4 μg/ml;and IGF-II F-20, sc-7435 at 0.4 μg/ml) were incubated overnight at 4°C. Negative controls were incubated with either goat IgG (sc-2028;Santa Cruz), at the same concentration as the target antibody, or with no antibody (diluent only). Goat anti-rabbit biotinylated secondary antibody (DAKOGlostrup, Denmark) was used before incubation with streptavidin/horseradish peroxidase-conjugated (DAKO) and was colour developed with liquid 3, 3'-diaminobenzidine (DAB)(DAKO) for 2 to 5 minutes. Sections were counterstained with a 1:10 dilution of Lillie-Mayer Haemotoxylin (AHLMAustralian Biostain Pty. Ltd, Tralagon, VIC, Australia).

### Quantitative RT-PCR analysis

Quantitative real-time PCR was used to quantify *INS* and *IGF2* expression in approximately 61 to 65 different mammary gland samples:20 to 22 from phase 1 of lactation (during pregnancy), and 25 to 27 from phase 2A (day 0 to day 100 of lactation), 11 to 12 from phase 2B (day 100 to 200 of lactation) and 4 to 5 from phase 3 (day 200 to day 350 of lactation). There were at least three samples (usually four or five) at each time point. For each sample, 800 ng total RNA was reverse transcribed using the SuperScript III First Strand Synthesis System for RT-PCR kit (Invitrogen). All primers crossed intron–exon boundaries (see Additional file 3). The *INS* probe, labelled at the 5^′^ end with 6-carboxy fluorescein, was designed using Biosearch Technologies Real Time Design software (Novato, CA, USA). *18 S* and *β-ACTIN* were used as reference genes. Reactions were performed in triplicate in 20 μl volumes consisting of 1 × Brilliant® II QRT-PCR Master Mix (Stratagene, Agilent Technologies, Santa Clara, CA, USA), 0.3 μM forward and reverse *INS* primers, 0.2 μM MTR probe, and 1 μl cDNA template. The *18 S* primers and probe were used at approximately 0.25 μM. *IGF2* PCRs were performed as above but used FastStart Universal SYBR Green Master (Rox) (Roche Products Pty Limited Dee Why, NSW, Australia), and 0.4 μM forward and reverse *IGF2* or 0.3 μM forward and reverse *β-ACTIN* primers were added without a MTR probe. Primer, probe, and cDNA concentrations were optimised in preliminary experiments.

Real-time PCR was carried out in an Stratagene Mx3000PTM Sequence Detector (Integrated Sciences, Chatswood, NSW, Australia) using the following conditions: 95°C for 10 minutes, followed by 50 (*INS*) or 45 (*IGF2*) cycles at 95°C for 30 seconds, 63°C (*INS*) or 61°C (*IGF2*) for 1 minute and 72°C for 30 seconds. A pancreas (*INS*) or liver (*IGF2*) sample triplicate and a negative template (water) triplicate were included on each plate as a calibrator and negative control, respectively. The data were analysed in Microsoft Excel and R [86]. The amplification efficiency was calculated from the standard curve and the cycle threshold values were corrected [87].

### Allelic expression analysis

Direct sequencing of cDNA (as described above) was used to confirm allelic expression of animals heterozygous at one or both polymorphic sites. Approximately 0.5 to 1 μl template was used with 0.2 μM each primer for *INS* or *IGF2* with either GoTaq Green Master Mix (Promega) or TaKaRa Ex Taq HS (Takara Bio Inc. Otsu, Shiga, Japan). RT-PCR cycles consisted of 94 or 96°C for 1 minute, followed by 30 to 35 cycles of 30 seconds at 94 or 96°C, 1 minute at 63°C, and 30 seconds at 72°C, and a final extension at 72°C for 5 minutes. PCR products from cDNA and genomic DNA were resolved by gel electrophoreses and the bands were extracted (QIAquick Gel Extraction Kit; Qiagen). The purified product was then sequenced using the Seq primers, designed for optimal sequence results (see Additional file 3). Sequences were assessed using FinchTV (v.1.3.1) DNA sequence chromatogram trace viewer software. The relative peak height for each allele indicates biallelic (equal peak heights) or imprinted (unequal peak heights) expression.

### 5^′^-Rapid amplification of cDNA ends

To acquire the full-length transcript for tammar *INS* we performed 5^′^-RACE, using both the SMARTer RACE cDNA Amplification Kit (Clontech, Mountain View, CA, USA) and 5^′^ Rapid Amplification of cDNA Ends, version 2.0 (Invitrogen). GSP1 primer was used to synthesise first-strand cDNA from 5 μg total RNA. GSP2 was used in conjunction with the provided Abridged Anchor Primer to amplify the 5^′^ end of the transcript. PCR products were cloned using a pGEM®-T Easy vector and JM109competent cells (Promega). Plasmids were purified using Wizard® Plus SV Minipreps DNA Purification System (Promega) and sequenced.

### Methylation analysis

Using MethPrimer [69], 10 CpG sites were identified upstream of exon1. Then 1 μg DNA was treated with a sodium bisulphite solution at 50°C for 4 hours before ethanol precipitating and eluting in 50 μl tris-EDTA buffer. As a template we used 20 ng of bisulphite treated DNA with 0.2 μM each bisulphite primer in a TaKaRa Ex Taq HS 25 μl reaction. A 3-minute extension time in the PCR thermal cycling was used to reduce PCR bias by permitting the polymerase to read through CG-rich regions. As no SNP was available to test for experimental biases at the *TH-INS* TSS, a solution containing 5 mg/mlBSA and 5% glycerol was added to the PCR reactions as a denaturant to reduce PCR bias during amplification [88]. PCR products were cloned as described above and sequences were analysed using the Quma quantification tool for methylation analysis [89]. A G/A SNP site was located in the CGI downstream of *TH-INS* TSS and a G/T SNP was located in the *INS* TSS region (Figure 6).

## Abbreviations

BSA: Bovine serum albumin; CGI: CpG island; DMR: Differentially methylated region; GSP: Gene-specific primer; IGF: Insulin-like growth factor; *INS*: Insulin gene; PCR: Polymerase chain reaction; *Peg3*: Paternally expressed gene 3; 5^′^-RACE: 5^′^-rapid amplification of cDNA ends; RT: Reverse transcriptase; SNP: Single nucleotide polymorphism; TH: Tyrosine hydroxylase; TSS: Transcription start site; UTR: Untranslated region.

## Competing interests

The authors declare that they have no competing interests.

## Authors’ contributions

MBR conceived the idea. MBR, JMS, AJP and GS designed the experiments. JMS performed the experiments. JMS, SS, MBR, GS and AJPanalysed the data. JMS, MBR, SS, GS and AJP wrote the paper. All authors read and approved the final manuscript.

## Supplementary Material

Additional file 1 ** Insulin immunolocalisation positive controls.** The immunolocalisation of insulin in the pancreas and placenta, providing positive controls to verify authentic protein recognition. Click here for file

Additional file 2 ** Genotyping and*INS*imprinting in the tammar wallaby.** Additional tables and figures that display the number and proportion of *INS* alleles within a population and extra sequence chromatographs of *INS* in the mammary gland. Click here for file

Additional file 3 ** Primers used in this study.** A table listing all of the primers used in this study. Click here for file
