# Supplementary material for: Selected imprinting of INS in the marsupial
Source: Epigenetics Chromatin. 2012 Aug 28;5:14. doi: 10.1186/1756-8935-5-14 (PMC3502105; doi:10.1186/1756-8935-5-14)
Supplement: Additional file 1 — Insulin immunolocalisation positive controls. The immunolocalisation of insulin in the pancreas and placenta, providing positive controls to verify authentic protein recognition. [file 1756-8935-5-14-S1.pdf]

Additional file 1 - **Insulin immunolocalisation positive controls**

Selected imprinting of *INS* in the marsupial

Jessica M Stringer, Shunsuke Suzuki, Andrew J Pask, Geoff Shaw, Marilyn B Renfree

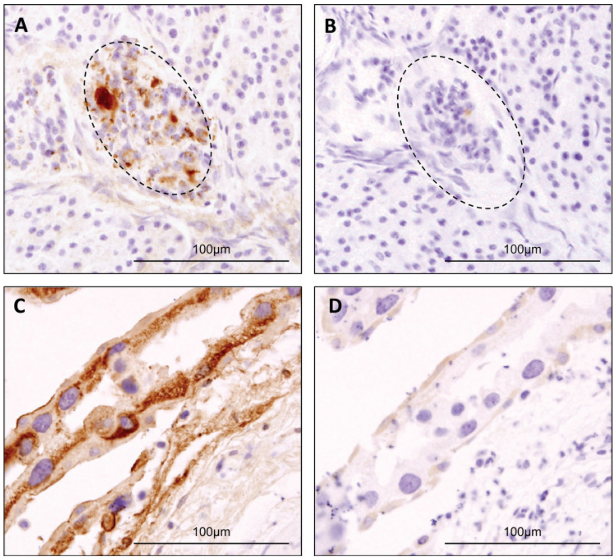

Figure S1. Immunolocalisation of insulin protein in the pancreas and placenta.
(A) Pancreas. Insulin (brown staining) was localised in the cytoplasm of the cells of the islets of Langerhans. (B) IgG negative control. (C) Placenta. Insulin was detected in the bilaminar yolk sac placenta as previously shown(Ager et al. 2007). (D) IgG negative control.
