# Supplementary material for: Selected imprinting of INS in the marsupial
Source: Epigenetics Chromatin. 2012 Aug 28;5:14. doi: 10.1186/1756-8935-5-14 (PMC3502105; doi:10.1186/1756-8935-5-14)
Supplement: Additional file 2 — Genotyping andINSimprinting in the tammar wallaby. Additional tables and figures that display the number and proportion of INS alleles within a population and extra sequence chromatographs of INS in the mammary gland. [file 1756-8935-5-14-S2.pdf]

Additional file 2 - **Genotyping and INS imprinting in the tammar wallaby**

Selected imprinting of *INS* in the marsupial

Jessica M Stringer, Shunsuke Suzuki, Andrew J Pask, Geoff Shaw, Marilyn B Renfree

Table S1. *INS* alleles.

Site 1	Site 2	Allele
G	C	1
G	T	2
A	T	3
A	C	4

There are two SNP sites in the tammar *INS* gene (site 1 G/A and site 2 C/T) and 4 possible different sequence combinations or alleles.

Table S2. Proportion of *INS* alleles in a population of 32 animals.

Allele	1	2	3	4		
	f, m	f, m	f, m	f, m	Freq	%
**1**	7, 5	5, 6	2, 2[a]	0, 0	39	61
**2**		1, 1	1, 1	0, 0	17	26.5
**3**			1, 0	0, 0	8	12.5
**4**				0, 0	0	0

[a] animals that polymorphic at both SNPs were cloned and shown to have alleles 1&3.

Thirty-two animals (17 females, 15 males) were randomly selected from the Melbourne tammar wallaby breeding colony and genotyped via direct sequencing. The number of females (f) and males (m) from the population is indicated for each possible allele combination. The frequency of appearance of each allele in the population was calculated and presented as a percentage of the total number of alleles (64). Allele 1 was the most common allele occurring at a frequency of 61% and is likely to be the original *INS* allele. A mutation at site 2 (C to T) would produce allele 2 which is the next most common allele. A second mutation on allele 2 at site 1 (G to A) formed allele 3. As the SNPs are so close together, the chance of recombination is minimal, thus explaining the absence of allele 4.

Table S3 - Monoallelic expression of *INS* in the mammary gland of 7 individuals found to be heterozygous.

Animal	Stage of Pregnancy/Lactation (days)	Alleles	Allele Expressed
A	14 of pregnancy	1&3	1
B	19 of pregnancy	1&3	1
C	25 of pregnancy	2&3	2
D	25 of pregnancy	1&2	1
E	8 of lactation	1&2	1
F	104 of lactation	1&2	1
G	183 of lactation	1&2	1

Imprinting in the mammary gland was detected in all stages of lactation examined: Phase 1 (pregnancy); Phase 2a (early lactation) and Phase 2b (mid- to late lactation). Of seven heterozygous adult female animals six carried allele 1 in combination with allele 2 (n=4) or 3 (n=2). All six females had clear monoallelic expression of allele 1 in the mammary gland. One animal carried allele 2 and allele 3 and had clear monoallelic expression from allele 2. Allele frequencies in the heterozygotes were consistent with the population allele frequencies calculated in Table S2.

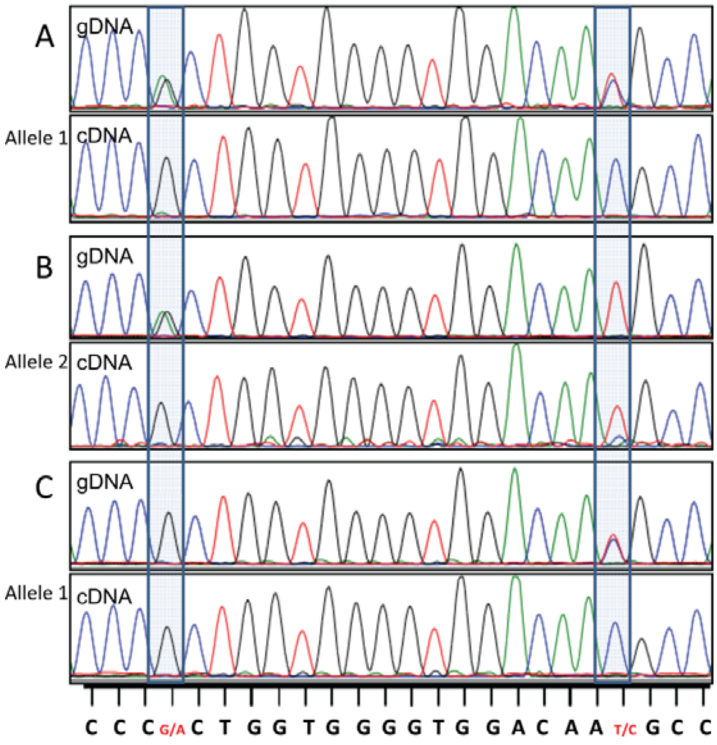

Figure S2. ***INS*** **Sequence chromatographs for analysis of allelic expression in the mammary gland**.

Direct sequence analysis for *INS* in the mammary gland showed three distinct alleles identified in the seven heterozygous female animals (see Table S3). Chromatogram traces of genomic DNA (gDNA) and complementary DNA (cDNA) from the mammary gland. (A) Representative of animals A and B. gDNA contains allele 1 and allele 3, both animals only expressed allele 1 (cDNA). (B) Chromatogram traces for animal D. gDNA contains allele 2 and allele 3 while the mammary gland only expressed allele 2. (C) Animals C, E, F and G carried allele 1 and allele 2 (gDNA) and expressed allele 1(cDNA). These results indicate that allele 2 can be both active and inactive. While allele specific expression due to cis-acting regulatory polymorphisms cannot be ruled out without a reciprocal cross, the clear monoallelic expression of two different *INS* alleles in the mammary gland, along with imprinted expression in the liver and placenta, suggests that *INS* may be imprinted in the mammary gland.
