# Supplementary material for: Selected imprinting of INS in the marsupial
Source: Epigenetics Chromatin. 2012 Aug 28;5:14. doi: 10.1186/1756-8935-5-14 (PMC3502105; doi:10.1186/1756-8935-5-14)
Supplement: Additional file 3 — Primers used in this study. A table listing all of the primers used in this study. [file 1756-8935-5-14-S3.pdf]

Additional file 3 - **Primers used in this study**

Selected imprinting of *INS* in the marsupial

Jessica M Stringer, Shunsuke Suzuki, Andrew J Pask, Geoff Shaw, Marilyn B Renfree

Gene	Primer	Primer sequence (5' to 3')	Reference
INS	cDNA exon2 Fw	TGACATGGCTCCCTGGTTGGC	a
	cDNA exon2/3 Rv	TCCGGCCTCACCCAACAGTG	
	cSeq RV	GGCGTGCTTTGGGAGTATAG	a
	gDNA Fw	GGACAGGAACCTGAGACTGG	[57]
	gDNA Rv	CTTGACATGGCTCCCTGG	
	gSeq RV	ACAGTGGCTGCTCAGCATC	a
	GSP1	(*INS* cSeq RV)	a
	GSP2	CCCCACACACCAGGTACAA	
	QPCR Fw	GCTGAGCAGCCACTGTTGGG	a
	QPCR Rv	TGGCCGCTAGTTGCAGTAAGTC	
	Probe	CAGTATCTGTTCCCTTTACCAGCTGGA	
	gDNA TSS Fw	GGTACCTCAGCTGTTATTGAGGAG	a
	gDNA TSS Rv	AAGACATGGATATAGAGGGGGCATA	
	B/S Fw	GGTATTTTAGTTGTTATTGAGGAGAG	a
	B/S Rv	ACCACCCATAAATACCTAACTTCTC	
	cDNA exon1 Fw	GCTCCATCTTCTTTTCTCACCTGTCTCT<br>(*INS* cDNA exon2/3 Rv)	a
TH-INS	cDNA Fw:	TTCGTGTCAGAGAGCTTTAGCGATG<br>(*INS* cDNA exon2/3 Rv)	a
	gDNA CGi Fw	AAGAGGTTTGGTGACTTCCTGTCCT	a
	gDNA CGi Rv	TTAGCTCATAGCGTTGAGAGCATGG	
	gDNA TSS Fw	CCTCACCTGGACTCTGGTCTGACT	a
	gDNA TSS Rv	CCAGGCCAACAGTGAGGGTTC	
	B/S TSS Fw	TTTTTTGTTTGAGGAGTTAGAGATT	a
	B/S TSS Rv	ATATCCATACCAAAACCTACCAAAC	
	B/S CGI Fw	GGTTTTTGTTTTTTTTGTGTGTTTT	a
	B/S CGI Rv	TAAAACTCATCCTAAACCCCTTACA	
IGF2	Fw	AAGTAACCTGGGTTCCTGCC	[68]
	Rv	CTCTTCTGCCGAAGGATGAC	a
	Seq Fw	GAGAGGGCCTCGCTACCAAC	a
	QPCR Fw	TGGGTTTCCCAATGAAGAAG	a
	QPCR Rv	GATTCAGGCGACGACTCAC	
B-ACTIN	Fw	TTGCTGACAGGATGCAGAAG	
	Rv	AAAGCCATGCCAATCTCATC	
18S	Fw	CGGCTACCACATCCAAGGAA	
	Rv	GCTGGAATTACCGCGGCT	
	Probe	TGCTGGCACCAGACTTGCCCTC	
GAPDH	Fw	CCTACTCCCAATGTATCTGTTGTGG	
	Rv	GGTGGAACTCCTTTTTTGACTGG	

Primers were designed for this study[a] using Primer3 (v. 0.4.0) [84] or derived from the references shown.
